# Aposematic signalling in prey-predator systems: determining evolutionary stability when prey populations consist of a single species

**DOI:** 10.1007/s00285-022-01762-y

**Published:** 2022-07-23

**Authors:** Alan Scaramangas, Mark Broom

**Affiliations:** City University of London, London, EC1V 0HB UK

**Keywords:** Evolutionary game theory, Local ESS, Continuous traits, Chemical defences, 92B05

## Abstract

Aposematism is the signalling of a defence for the deterrence of predators. We presently focus on aposematic organisms that exhibit chemical defences, which are usually signalled by some type of brightly coloured skin pigmentation (as is the case with poison frog species of the *Dendrobatidae* family), although our treatment is likely transferable to other forms of secondary defence. This setup is not only a natural one to consider but also opens up the possibility for rich mathematical modelling: the strength of aposematic traits (signalling and defence) can be unambiguously realised using variables that are continuously quantifiable, independent from one another and which together define a two-dimensional strategy space wherein the aposematic behaviour of any one organism can be represented by a single point. We presently develop an extensive mathematical model in which we explore the joint co-evolution of aposematic traits within the context of evolutionary stability. Even though empirical and model-based studies are conflicting regarding how aposematic traits are related to one another in nature, the majority of works allude to a positive correlation. We presently suggest that both positively and negatively correlated combinations of traits can achieve evolutionarily stable outcomes and further, that for a given level of signal strength there can be more than one optimal level of defence. Our findings are novel and pertinent to a sizeable body of physical evidence, which we discuss.

## Introduction: an overview of aposematic signalling

Aposematism is a complex biological process that involves the mobilisation of a defensive and a signalling trait and has revealed challenges of considerable magnitude to theorists and experimentalists alike. Out of the many unanswered questions in aposematism, an obvious one stands out: how should the defence and the signalling of that defence co-evolve? We explore new answers to this question by building on those already provided in Broom et al. (2006) and use these to deepen our understanding of more curious instances in nature that could not be accounted for from previous models. We should note that aposematism is manifest in a vast array of physical systems among which model-based approaches may vary. The article at hand is structured such that the context within which we seek to answer questions is detailed first (Sects. 1 and 2), while the specific objectives are defined (Sect. 2), addressed (Sect. 3) and discussed therein (Sect. 4). This introductory section is two-fold: the first subsection provides details from the underlying biology as in Broom et al. (2006), while the second discusses previous and more contemporary model-based approaches in aposematism (their efficacy to deepen our understanding of empirical studies is further discussed in Sect. 4).

### Notes from the underlying biology

Organisms of all types exhibit defence mechanisms that are deployed to prevent potential predators from mounting attacks. These exist in a variety of guises, are manifested in a range of different ways, trigger the senses differently and pose different fitness advantages to those who deploy them (Ruxton et al. 2018). Defences can either be permanently present (*static constitutive*) in the prey individuals that acquire them or can be deployed during conflict (*induced*). While the latter have lower costs associated with maintenance and are effective against attacks that take place over a longer time period, these are not considered in our paper as they are generally less effective against the fast-paced and potentially lethal attacks which we consider herein. Prey defend themselves against such attacks by reducing the likelihood of encounter (*primary defences*) and of the probability that an attack results in death as well as by reducing the probability that similar-looking prey are attacked in the future (*secondary defences*) - see Broom et al. (2006).

Primary and secondary defences are used in conjunction to form more composite forms of defence, an important example of this being *aposematic defence*, which we study presently (in most populations aposematic individuals are rare compared with their camouflaged counterparts - see Santos et al. (2003), Vences et al. (2003) and Ruxton et al. (2018)). In most discussions, aposematic signalling is understood as a signal that informs potential predators of the existence of a defence that may not be readily detectable by predators. Chemical defences are a typical example of this as they are retained within the body and are usually not detectable to the predator unless it has made physical contact with the prey. Indeed, chemical defences might be the classical example of defences that can be detected after an attack has been attempted (although behavioural defences such as the ability flee or fight back also exist).

It is argued that both predators and prey can benefit from honest signalling of chemical defences if there are costs to both parties associated with prey capture prior to detection of defences (such as time and energy invested in chasing and fleeing, and/or risk of injury). Other defences, however, can be easily detectable by predators at a distance without the predator making physical contact with the prey (typical examples being mechanical defences such as a thick shell or spines). It is still possible that signalling of such defences can be selected because they aid both predator and prey. In this case, the signal is likely to function so that it draws the predator’s attention to the defence therefore reducing costs to both parties that might occur because the predator failed to notice the defences.

Broadly speaking, aposematic signals are phenotypes with three additional properties (Ruxton et al. 2018). First, they are paired (at least in the mind of a predator) with some form of secondary defence (e.g. bright skin pigmentation is paired with unpalatable toxins). That is, the predator’s response to the signal will be linked to a cognitive association the predator has formed between the signal and the defence or other aversive trait that makes prey unprofitable to predators. Second, they have evolved as signals through natural selection and are therefore effective in altering predator behaviour so that it favours prey survival (e.g. fewer recognition errors, enhanced wariness, accelerated learning and decelerated forgetting). Third, these alone can act as a primary defence mechanism in the sense that it is deterring for predators (through *learned aversion* - see Broom et al. 2006) and thus reduces the probability that an attack is mounted.

There is a considerable volume of empirical evidence suggesting that chemical defences incur fitness costs (reductions in growth, adult size or fecundity - see Darst et al. (2006) or Zvereva and Kozlov (2016) and the bibliography therein) most likely associated with the costs of synthesising and/or acquiring (e.g. through dietary modifications - see Daly (2003) or Darst et al. (2005)) and/or storing toxins. Although it may be that there is no evidence of costs being incurred at all in some cases, as is the case with *Diprion pini* larvae discussed in Lindstedt et al. (2011) for example. It is also conceivable that fitness costs are less apparent among prey whose defences have successfully evolved beyond those primary stages: Tarvin et al. (2017) observe that numerous species of the *Dendrobatidae* family undergo significant self-poisoning upon acquiring toxins but whose impact diminishes through ever-increasing amino acid replacements in toxin-binding sites.

Presently and in Broom et al. (2006) it is assumed that toxins are costly such that investment in these involves a trade off in fecundity, while a similar but less explicit effect is seen with investment in conspicuousness. The setup described thus far opens up the possibility to study aposematism systematically and within a game-theoretical framework (detailed in Sect. 2) - a task that is achieved successfully and without sacrificing biological plausibility. The specific objectives are to understand *(a) how conspicuous and (b) how well-defended should a prey individual be so that a population made up entirely of one type can maintain their composition?* In the following subsection we review the first major contribution in this direction (Leimar et al. 1986), compare it with the model of Broom et al. (2006) and hence motivate the relevance of our own insight into this challenge.

### Game-theoretical modelling of aposematism

Aposematic traits necessarily co-evolve so that primary defences (e.g. camouflage) are traded off in favour of a conspicuous appearance, which may enhance predator’s learned aversion to defended prey. But how these traits co-evolve to optimise fitness is relatively unknown compared with our knowledge of the costs that these incur to individuals who deploy them. Leimar et al. (1986) were the first to study this problem and their model is especially important in this regard, not only due to its focus and its level of detail (see predator psychology, prey behaviour and population structure) but also due its novel game-theoretical approach. Indeed, predator attack probabilities are calculated through varied rates of learning and sensory generalisation, while the effectiveness of prey unprofitability is evaluated in terms of individual survival, effects on predator learning rates, cost of investment and of the associated optimal conspicuousness. The model predicts that for given level of conspicuousness there can be a unique (nontrivial) optimal value of unprofitability, which can increase if either survival rates increase and/or if increased prey unprofitability is linked with increased learning rates.

The second, game-theoretical contribution to the theory of aposematism we discuss came twenty years later and is due to Broom et al. (2006). The focus of the paper at hand is to uncover implications of this model that had not been seen upon its publication (neither in Broom et al., 2008) and which may help us gain deeper understanding of an important body of physical evidence on aposematism in nature. The details of this model are left for the following section, although we presently note the following. Broom et al. (2006) consider a large population of prey (among which aposematism is already established) that maintain dynamic equilibrium with a large population of predators such that the level of predation pressure remains fixed. The aposematic behaviour of any one prey item is realised using two-by-one vector (strategy) containing continuously quantifiable, positive entries that represent the signalling strength and level of defence of that item (the original model considers a three-by-one vector). Using functional forms that detail prey-predator interaction and prey sensitivity to traits, the notion of fitness/payoff is defined and interpreted as the number of offspring produced per given life cycle. The related concept of an *Evolutionarily Stable Strategy* (ESS) is defined and constitutes a particular combination of traits which when assumed by almost all members of the prey population tends to be retained by successive generations (in absence of drift). Broom et al. (2006) argued that strategies of this type should be contained within an increasing continuum, such that to a given level of signal strength is assigned a unique optimal level of defence. We presently show that these restrictions need not apply and hence draw interesting connections with the underlying biology.

The model of Leimar et al. (1986) is in a sense complementary to the model of Broom et al. (2006) in that their overall focus (co-evolution of aposematic traits) and their detailed game-theoretical approaches are common but their individual aims vary. In particular, Leimar et al. (1986) aims to study the origins of aposematism and considers a set of initially naïve predators, whose attack probabilities are maximum before they have their first encounter ($G^{(0)}(x) = e(x)$, with *x* representing the level of prey unprofitability). Over successive prey encounters (labelled by *n*) these attack probabilities continuously decreases according to an inhibitory gradient $h(x,x_1,y_1)$ (with $x_1$ being the unprofitability of encountered prey and $y_1$ its conspicuousness), so that

1 $$\begin{aligned} G^{(n)}(x,x_1,y_1) = e(x)[1-h(x,x_1,y_1)]^n. \end{aligned}$$

This equality suggests that repeated encounters affect (decrease) the level of predation pressure. Notably, such a predator strategy would be unstable in the context of Broom et al. (2006), which instead considers a regime of fixed predation pressure, which may have been reached by learning and/or genetic inheritance and is carefully maintained through a balanced mix of young and old individuals in overlapping generations. It is thus suggested that the model of Leimar et al. (1986) could pertain to more short-lived (such as seasonal) predators and the model of Broom et al. (2006) to longer-lived ones. A final remark is that the type of solution considered in Leimar et al. (1986) is not the same as the local ESSs that we consider presently (see Sect. 2.2) or in Broom et al. (2006) and indeed the latter should be consulted for a more elaborate comparison of the two models.

It should be noted at this stage that there are a number of studies in which it is incorrectly claimed that Leimar et al. (1986) suggest that optimal levels of conspicuous and defence are negatively correlated across populations. This is most recently done in Summers et al. (2015), which constitutes an otherwise indispensable review of empirical and model-based approaches on *honest signalling* and indeed one that we consult in the discussion section of this article. As it happens, the model of Leimar et al. (1986) does not consider signal strength at all, it only compares a non-signalling (camouflaged) phenotype to a signalling phenotype. In particular, it is argued that if the signalling phenotype is associated with a reduced rate of attack by predators (perhaps through learned aversion) such that the non-predatory cost of producing toxins increases with its effectiveness in reducing predator attack rates then the optimal strategy may be for the signalling phenotype to invest less in costly toxins than the non-signalling phenotype. The possibility of a true, negative correlation between signal strength and level of defence within a causal game-theoretical framework is conceived for the first time presently and forms a novel extension to the predictions of Broom et al. (2006).

Broom et al. (2006) were the first to study the co-evolutionary stability of aposematic traits in the sense of a true ESS and subsequent explorations of the model (Ruxton et al. 2007a; Broom et al. 2008; Teichmann et al. 2014) have been successful in generating better understanding of its underlying implications. Operating within the framework of Broom et al. (2006) we presently recover solutions of the type that would not be expected therein and hence suggest that optimal combinations of aposematic traits can follow a more complex relationship than what was originally expected. Interestingly, we deduce that optimal levels of conspicuousness and defence can be both positively and negatively correlated and that for given level of conspicuousness there can be more than one optimal level of defence. We have made the critical observation that the monotonicity of a continuum of ESS solutions (of the type that is predicted in Broom et al. (2006) and more explicitly displayed in Broom et al. 2008) is governed by the *Implicit Function Theorem* (in ${\mathbb {R}}^2$). Examples of evolutionarily stable continua are demonstrated (see Sect. 3) using functional forms that are both biologically plausible and yet carefully chosen to negate the premise that increased conspicuousness necessarily leads to increased rates of attack. Indeed doing so has brought forward insight into the model that is novel and has led us to revise some restrictive assertions that were made upon its publication.

## Model description and evolutionary stability

We begin this section by introducing the model of Broom et al. (2006) in its most general setting, whereby prey individuals are allowed to assume any strategy and hence proceed by setting the scene for what will constitute our main focus henceforth, namely the study of evolutionary stability. Within the latter, we seek to find a strategy such that if almost all of the prey population were to play it (residents) then these could not be invaded by a minority (of mutants) playing a slightly different (local) strategy. Such a strategy is said to be (locally) evolutionarily stable and when played by almost all individuals of the prey population, successive generations tend to retain it (in absence of drift). Upon describing the model in terms of the relevant quantities, parameters and functional forms (the notion of *payoff* included), the conditions for (local) evolutionary stability are presented, as is their novel connection with the *Implicit Function Theorem*.

### Unrestricted prey strategies

We consider a population of prey of a certain species who occupy some habitat. Assume that the habitat can be partitioned into effectively infinite, non-overlapping localities consisting of approximately *N* prey, where *N* is taken to be large. In addition, assume that each locality is visited by *n* predators, who visit a single locality only. Implicit in this layout is that there is a uniform territorial division of the habitat among the predators so that each locality can be perceived as the territory of a specific number of *n* predators (*n* is too taken to be large). It is also generally assumed that the overall population of prey and predators are in dynamic equilibrium so that in any one locality predation has no effect on the relative sizes *N* and *n*.

Within each locality prey are labelled by the index *i* , such that $\{i=1,2\ldots ,N \}$ and defend themselves against predators by investing in chemical defences of *toxicity* $t_i \ge 0$, which are advertised by bright skin pigmentation of *conspicuousness* $r_i\ge 0$ (totally *cryptic* prey have $r_i=0$). The aposematic traits are naturally independent from one and other and are thus represented by the vector $(r_i,t_i)$, which denotes the *strategy* of prey individual *i* - the original model description in Broom et al. (2006) involves 3-vector strategies that include prey *colouration* $\theta _i \in [0, 2\pi )$, which we presently omit.

Prey item *i* reproduces with *fecundity* rate $$F = F(t_i)$$ (*F* is a declining function of $$t_i$$, indicating that investment in toxins is costly), dies of causes other than predation at some fixed $$background mortality rate $$ $$\lambda $$ (we set $$\lambda = 0$$ in our analysis) and defend themselves against predators by acquiring aposematic traits $$(r_i,t_i)$$. Upon detecting prey, predators can decide to mount an attack, which may or may not lead to capture. The *detection rate* $$D(r_i)$$ is an increasing function of $$r_i$$ and tends to unity as prey conspicuousness assumes arbitrarily large values - indeed an interval of unit time can be defined as the time taken for a very bright prey item to be detected by a predator - while $$D(0)=d_0>0$$, since even fully-cryptic prey can be detected. The probability that a mounted attack results in capture is given by $$K=K(t_i)$$, where *K* is declining with $$t_i$$, indicating that more defended individuals are harder to capture. A detected individual will be attacked with probability $$Q = Q(I_i)$$, where $$I_i$$ represents the *average aversive information* that the average predator has on item *i*. Naturally, it is assumed that *Q* is a declining function of $$I_i$$ and such that $$Q(I_i)=1$$ for $$I_i\ll -1$$ and $$Q(I_i)\approx 0$$ for $$I_i \gg 1$$.

Predators assign $I_i$ to individual *i* by comparing it to a certain (weighted) base-line level of aversive information, which is generated through encounters with the prey population, such that2It is understood that any one locality is quite sizeable (i.e. *N* is large) and is visited by a group of predators *n*, who visit this locality only. Tacit in this discussion is that it is (usually) not possible for any one predator to experience every single prey item within its life-cycle; rather, we assume that predators experience the locality collectively and the aversiveness of their experiences is shared equally among them (see factor of 1/*n* in (2)). Even though as a collective, predators have complete experience of the locality, their perceived aversiveness of a particular individual *i* is not drawn directly by their experience of it, but through successive experiences with its neighbours (notice that the sum in (2) excludes *i*, but includes the remaining locality).

An important assumption of our model is that predators learn quickly, so that most of their life they impose mortality based on their understanding of prey traits gathered during a short investigative learning phase early in life. Since learning involves only a short fraction of the predator’s lifetime it can be mostly ignored from the prey perspective. Indeed, the overwhelming majority of prey mortality is caused by experienced predators, who have completed the learning process described in (2). A plausible example of a system that this model might best describe are insectivorous birds, who might consume hundreds of insects a day and live for several years providing the prey population is consistent under that timescale (this might suggest a tropical rather than temperate region). Under such a description we might expect that birds learn about prey that they encounter on a timescale of days, which is notably shorter than their lifetime.

The similarity function3describes how predators perceive the *visual similarity* of different-looking prey. While the (generalised) similarity function  of (3) (denoted with calligraphy) is naturally bi-variate, in Broom et al. (2006) and indeed for the remainder of this discussion we will treat this a as a uni-variate function *S* of the Euclidean distance separating their visual appearance. In particular we impose that4The uni-variate function S on the RHS of (4) is assumed to be $\mathrm{{C^2}}$ and have the additional four properties. i) $S(|r_i-r_j|)=1$ if and only if *i* and *j* are identical ($r_i=r_j$); ii) $S(|r_i,r_j|)=0$ if and only if *i* and *j* are very dissimilar ($r_i \gg r_j$ or $r_j \gg r_i$); iii) *S* is non-increasing with $|r_i-r_j|$ (perceived visual similarity does not increase as signals vary increasingly in strength - see iv) and subsequent discussion for the behaviour of *S* near the origin); iv) the similarity function is not flat-peaked at the origin ($S^{\prime }(0) \ne 0$) . This last restriction on *S* is more controversial than the previous ones and imposing this depends on our interpretation of the predator’s cognitive abilities and psychology.

With a similarity function that is peaked at the origin the predator is sensitive to small differences in the conspicuousness of individuals so that even a small deviation between individuals in conspicuousness is associated with a drop in perceived similarity. In contrast, a similarity function that is flat at the origin implies that the predator generalises strongly between individuals that differ slightly in conspicuousness and thus perceives two individuals that vary only slightly in conspicuousness as very similar. We note that since the slope is non-zero away from the origin, whilst predators cannot distinguish easily between those types that they are used to encountering, they can tell apart small visual differences, relative to the common type, between types that they are not used to encountering, and we thus consider the peaked function preferable. We should remark that our results would hold for similarity functions that are peaked in the manner described above and would not hold if the maximum of the generalisation curve were smooth (like a normal curve) - see also Ruxton et al. (2007b). Restrictions on the shape of the generalisation functions are discussed in Balogh and Leimar (2005) in the context of the evolution of mimicry.

Predators find chemically-defended prey aversive and the experience of consuming them is measured by function $H=H(t_i)$, which is an increasing function of $t_i$ and is zeroed at a critical value of the toxicity $t_i=t_c$. Prey with $t_i<t_c$ are perceived as *negatively aversive* (attractive) by predators ($H(t_i)<0$), as their toxin level is not sufficient to outweigh the nutritional benefit received from consuming them, while prey with $t_i>t_c$ are non-trivially defended and are seen as *positively aversive* by predators ($t_i=t_c$ describes *neutrally aversive* prey). Lastly, $L=L(r_i)$ represents the rate at which encounters that have occurred can be recalled by predators and is a growing function of $r_i$ indicating that encounters with more conspicuous prey can be better recalled. We will take $L=D$ in our analysis, an idealisation describing *perfect predator recollection*. We include a list of symbols and their meanings for easy reference (see Table 1).

**Table 1:** The parameters and functions of the model

Symbol	Meaning
*r*	the conspicuousness of a prey individual
*t*	the toxicity of a prey individual
*N*	the size of the prey population
*n*	the size of the predator population
*D*(*r*)	the rate at which *r*-individuals are detected
*L*(*r*)	the rate at which *r*-individuals are detected and recalled
	the generalised (bi-variate) similarity function applied to individuals with appearances ${r_i}$ and ${r_j}$
*S*(*x*)	the uni-variate similarity function of individuals differing in appearance by *x*
*H*(*t*)	the aversiveness of prey individuals with toxicity *t*
$t_c$	the critical level of toxicity such that $H(t_c)=0$
*F*(*t*)	the fecundity of a prey individual with toxicity *t*
*K*(*t*)	the probability that an attacked *t*-individual is captured
*Q*(*I*)	the probability that a detected *I*-individual is attacked
*I*	the level of aversive information if an individual
$\lambda$	the prey background mortality rate (not due to predation)
*a*	the average relatedness of prey individuals in the population

Detection, attack and capture are independent events and thus the *predator-induced mortality rate* of item *i* is given by $P(Capture|Attack \,of\,i)\times P(Attack|Detection \, of\,i) \times (Rate\,\, of\,\, detection\,\,of\,\, i)= K(t_i)Q(I_i)D(r_i)$ and therefore the total mortality rate for *i* is $\lambda + D(r_i)K(t_i)Q(I_i)$. The payoff to item *i* playing strategy $(r_i,t_i)$ is a unitless number defined as the number of offspring it produces during its life-cycle. This can be understood in terms of the existing functional forms as the rate of reproduction (*F*) over the overall rate of death ($\lambda +DKQ$), whose reciprocal gives us the average life-cycle. In particular, we have

5 $$\begin{aligned} P(r_i,t_i) = \frac{F(t_i)}{\lambda + D(r_i)K(t_i)Q(I_i)}. \end{aligned}$$

It is important to note that the uni-variate functions *F*, *D*, *K*, *Q*, *H* and *L* are assumed to be of class $C^2$ over ${\mathbb {R}}$, while *S* is everywhere $C^2$ except at one point (a fact whose significance is discussed in the following subsection). This restriction is necessary and sufficient for expressing the conditions for evolutionary stability in the form of (15) through to (18) and for understanding how aposematic traits are correlated in terms of the Implicit Function Theorem (see (14)). This restriction also agrees with our perception of the physical world; it guarantees that organisms playing slightly different strategies also have very similar values for the different consequences of their strategies.

### Residents vs. mutants

The focus of our analysis is on the evolutionary stability of aposematic traits. We thus consider a resident-mutant setup of the prey population such that the majority of prey play some *resident strategy* $(r_1,t_1)$, while a much smaller fraction $\varepsilon \small {\ll } 1$ play some *mutant strategy* (*r*, *t*), which we restrict to be *local* to the resident strategy (contained in its neighbourhood in strategy space).

We now consider a certain *focal individual*, whose strategy $(\rho ,\tau )$ we leave unspecified (by setting $(\rho ,\tau )$ $\in \{(r,t), (r_1,t_1) \})$ and introduce imperfect mixing to the prey population by suggesting that in a small, finite number of localities there are *relatives* of the focal individual. We assume that these relatives are perfect copies of the focal individual and form * mutant colonies* that are assumed to be rare. Examples of colonies are seen in a number of different populations. In Cole (1946) the phenomenon of *“clumping of individuals into groups”* is described such that *each group may be relatively or entirely independent of all similar groups and, therefore, that these distributional units may be randomly distributed”*. A more elaborate discussion of the spatial distribution of insect populations can be found in Taylor (1984). Interestingly, examples of amphibian populations, which we generally regard in this paper may also form colonies (as is the case for *Polypedates leucomystax* frogs that are examined in Roy (1997)).

We introduce parameter *a* as a measure of the average local relatedness, which we define as the fraction of focal copies making up the colony over the total number of prey in the locality. Under this definition, parameter *a* is interpreted as a quantity measuring the concentration of relatives and implicit in this is that the focal individual breeds true. Almost all localities are empty of mutant colonies and in these there is no distinction between relatives and non-relatives as all individuals are residents. In very few however, mutant colonies exist and so a proportion *a* are distinct from the resident population. Furthermore, we assume that the background concentration of mutants in the habitat is carried through to the localities, such that if $a=0$ we expect on average to encounter $\varepsilon N$ mutants and $(1-\varepsilon )N$ residents. More generally, if *a* is non-zero we expect that there are $aN-1$ relatives making up the colony (excluding the focal individual) and $(1-a)N$ non-relatives of which $(1-a)\varepsilon N$ play the mutant strategy and $(1-a)(1-\varepsilon )N$ play the resident strategy. In light of (2) the perceived aversiveness of the focal individual reads

6 $$\begin{aligned} \begin{aligned} I^{\varepsilon }(\rho ,\tau )&= \frac{1}{n}(aN-1)L(\rho )H(\tau ) S(|\rho -\rho |) + (1-a)\varepsilon \frac{N}{n}L(r)H(t)S(|\rho -r|) \\&\quad + (1-a)(1-\varepsilon )\frac{N}{n}L(r_1)H(t_1) S(|\rho -r_1|). \end{aligned} \end{aligned}$$

In particular, the aversive information for the average mutant or resident is uncovered from (2) by setting the focal strategy $(\rho ,\tau )$ equal to (*r*, *t*) or $(r_1,t_1)$, giving

7 $$\begin{aligned} I^{\varepsilon }(r,t)= & {} a\frac{N}{n}L(r)H(t)\nonumber \\&+(1-a)\varepsilon \frac{N}{n}L(r)H(t) +(1-a)(1-\varepsilon )\frac{N}{n}L(r_1)H(t_1) S(|r-r_1|) \end{aligned}$$

and

8 $$\begin{aligned} I^{\varepsilon }(r_1,t_1)= & {} a\frac{N}{n}L(r_1)H(t_1)+(1-a)\varepsilon \frac{N}{n}L(r)H(t) S(|r-r_1|)\nonumber \\&+ (1-a)(1-\varepsilon )\frac{N}{n}L(r_1)H(t_1). \end{aligned}$$

In the limiting case $\varepsilon \rightarrow 0$ the above simplify to

9 $$\begin{aligned} I= & {} a\frac{N}{n}L(r)H(t) + (1-a)\frac{N}{n}L(r_1)H(t_1)({|r-r_1|}) \quad \text {and} \quad \nonumber \\ I_1= & {} \frac{N}{n}L(r_1)H(t_1), \end{aligned}$$

where for convenience we have adopted the notation $I(r,t) \leftrightarrow I$ and $I_1 \leftrightarrow I(r_1,t_1)$ for the mutant and resident information in the $\varepsilon \rightarrow 0$ limit. In keeping with the presentation of Broom et al. (2006) and indeed for reasons that are more clearly explained in Appendix IV

The mutant and resident payoffs thus read

10 $$\begin{aligned} P(r,t;r_1,t_1)= & {} \frac{F(t)}{\lambda + D(r)K(t)Q(I)} \quad \text {and} \quad \nonumber \\ P(r_1,t_1;r_1,t_1)= & {} \frac{F(t_1)}{\lambda + D(r_1)K(t_1)Q(I_1)}. \end{aligned}$$

In this context the resident strategy $(r_1,t_1)$ is an (local) *Evolutionarily Stable Strategy (ESS)* if it is stable against a mutant minority playing (*r*, *t*) in the locality of $(r_1,t_1)$ in strategy space. Indeed, in the $\varepsilon \rightarrow 0$ limit the condition amounts to the mutant payoff in (5) admitting a local maximum for $(r,t)=(r_1,t_1)$. In principle, the mutant payoff is a scalar function of four variables (two pairs of strategy vectors) and for given choice of resident strategy $(r_1,t_1)$ this is defined on the infinitesimal rectangle $(r_1-\delta r, r_1 + \delta r) \times (t_1-\delta t, t_1 + \delta t)$ ’centred’ at $(r_1,t_1)$. A local ESS is therefore a resident strategy $(r_1,t_1)$ that maximises the mutant payoff (over the rectangle) precisely at the ’centre’ of the rectangle $(r,t)=(r_1,t_1)$. It is important to remark that the conditions for maximising $P(r,t; r_1,t_1)$ over $(r_1-\delta r, r_1 + \delta r) \times (t_1-\delta t, t_1 + \delta t)$ cannot be deduced using the standard linearisation techniques from vector calculus, since $P(r,t; r_1,t_1)$ is not *r*-differentiable at $(r,t)=(r_1,t_1)$. In fact, after suitable positive scaling $\partial _{r}P(r,t; r_1,t_1)$ reads

11 $$\begin{aligned}&\frac{D^{\prime }(r)}{D(r)} -a \frac{N}{n}L^{\prime }(r)H(t)\frac{Q^{\prime }(I)}{Q(I)} \nonumber \\&\quad - (1-a)I_1\frac{Q^{\prime }(I)}{Q(I)}S^{\prime }(|r-r_1|)\left( -\mathbbm {1}_{(-\infty ,r_1)}+\mathbbm {1}_{(r_1,+\infty )} \right) , \end{aligned}$$

which is not defined at $r=r_1$ unless $S^{\prime }(0) =0$ - a possibility we exclude.

For the remainder of the discussion when referring to the mutant payoff $P(r,t;r_1,t_1)$ we may omit the second pair of arguments $(r_1,t_1)$, which leads to the shorthand notation $P(r,t;r_1,t_1) \leftrightarrow P(r,t)$. Indeed, this abbreviation carries through to the partial derivatives, such that

$$\begin{aligned} \partial _{t}P(r,t;r_1,t_1)|_{r=r_1,\,t=t_1} \leftrightarrow \partial _{t}P(r_1,t_1) \end{aligned}$$

and

$$\begin{aligned} \partial _{t_1}\big [\partial _{t}P(r,t;r_1,t_1)|_{r=r_1, \,t=t_1}\big ]|_{r_1=r_1^*,\,t_1=t_1^*} \leftrightarrow \partial _{t_1}\partial _{t}P(r_1^*,t_1^*). \end{aligned}$$

For the latter it should be remarked that differentiability is not a problem because $\partial _{t}P(r,t;r_1,t_1)|_{r=r_1, t=t_1}$ is everywhere differentiable with respect to the resident trait. In addition it can be shown that

12 $$\begin{aligned} \partial _{t_1}\partial _{t}P(r_1^*,t_1^*) = \partial _{t_1t}P(r_1^*,t_1^*) + \partial _{tt}P(r_1^*,t_1^*), \end{aligned}$$

and in particular that

$$\begin{aligned} \partial _{t_1t}P(r_1^*,t_1^*) \ne \partial _{t_1}\partial _{t}P(r_1^*,t_1^*). \end{aligned}$$

The quantity $\partial _{t_1t}P(r_1^*,t_1^*)$ on the LHS is the mixed partial derivative evaluated at $r=r_1=r_1^*$ and $t=t_1=t_1^*$. The quantity $\partial _{t_1}\partial _{t}P(r_1^*,t_1^*)$ on the RHS involves two steps: first taking the derivative with respect to the mutant trait and evaluating this at $r=r_1$, $t=t_1$. Second, differentiating this quantity (which is solely a function of the resident traits) with respect to the resident trait $t_1$ and evaluating the resident traits at $r_1=r_1^*$, $t_1 = t_1^*$. The quantity resulting from this two-step process is not the same as that on the LHS, which involves a single step. The consequence of this inequality is especially relevant when considering (14). The reader may consult the more detailed discussion of Appendix III.

Resident strategies $(r_1,t_1)$ are chosen from the boundary-inclusive, right-upper-hand plane $\{ (\rho , \tau ): \rho \ge 0, \tau \ge 0\}$ where the conditions for maximising mutant payoff (over its local vicinity) are different at the origin $\{(0,0)\}$ to what these are on the boundaries $\{(\rho ,0): \rho \ge 0 \}$, $\{(0,\tau ): \tau \ge 0\}$ or the interior $\{ (\rho , \tau ): \rho> 0, \tau > 0\}$. It is shown in Broom et al. (2006) that the region $\{ (\rho , \tau ): \rho > 0, \tau \le t_c\}$ does not contain local ESSs. On the aversive and conspicuous subregion $\{(\rho ,\tau ):\rho>0, \tau>t_c \} \subset \{(\rho , \tau ): \rho>0, \tau >0 \}$ the conditions for (local) evolutionary stability read

13 $$\begin{aligned} \partial _tP(r_1,t_1) =0, \quad \partial _{tt}P(r_1,t_1)\big |< 0, \quad \overset{\leftarrow }{\partial _r}P(r_1,t_1)>0 \quad \text {and} \quad \overset{\rightarrow }{\partial _r}P(r_1,t_1)<0.\qquad \end{aligned}$$

We should remark that non-aversive (cryptic) solutions are discussed in Appendix I and shown in Fig. 5. The partial derivatives with (left and right) arrows correspond to the *left* and *right* partial derivatives with respect to *r* of the mutant payoff function defined in (10) - see related discussion in Appendix III

Indeed, imposing that the mutant payoff function is maximised along the *t*-direction (at $t=t_1$) for fixed $r=r_1$ and likewise along the *r*-direction (at $r=r_1$) for fixed $t=t_1$ suffices to guarantee a local maximum at $(r,t)=(r_1,t_1)$. This can be explained by the discontinuity along the *r*-direction at $r=r_1$ (expressed in (11)) which in a sense ’dominates’ all other directional derivatives involving non-zero *r*-components. Resident strategies satisfying $\partial _{t}P(r_1,t_1)=0$ are called *t*-*equilibrium points* and can be seen as elements of the zero-level set of the map $(r,t)\mapsto \partial _{t}P(r_1,t_1)$ using the notation introduced above. Given that the mutant payoff is made up of combinations of at least $C^2$ functions, it follows that $\partial _{t}P$ is at least $C^1$ and thereby satisfies the conditions of the *Implicit Function Theorem* (IFT) in ${\mathbb {R}}^2$. This implies that if $(r_1^*,t_1^*)$ is a *t*-equilibrium point then there exists a smooth function *h* defined on the vicinity of $r_1^*$ with $h(r_1^*)=t_1^*$, whose slope is given by

14 $$\begin{aligned} -\frac{\partial _{r_1}\partial _{t}P(r_1^*,t_1^*)}{\partial _{t_1}\partial _{t}P(r_1^*,t_1^*)}. \end{aligned}$$

In Broom et al. (2006) it is argued that for most choices of biologically feasible functions the numerator in (14) is strictly positive whenever $t_1>t_c$ and self-consistent reasoning was given to support this. Although this result holds for the functions we consider in Sect. 3 (as well as for those considered in Broom et al. (2008)), it is not sufficient justification for ruling out the prospect of local ESSs that are decreasing with increasing conspicuousness as it does not account for the sign of the denominator. This point will be elaborated on in Sect. 3, where the $^*$ notation is dropped (strategies are chosen from the *t*-equilibrium curve by default).

For the case in which the only source of death for prey is due to predation ($\lambda =0$) and in which there is perfect recollection of encounters by predators ($L=D$) the ESS conditions of (13) with mutant payoff given as in (10) read

15 $$\begin{aligned}&\qquad \qquad \qquad \qquad \frac{F^{\prime }(t_1)}{F(t_1)} - \frac{K^{\prime }(t_1)}{K(t_1)}-aI_1\frac{Q^{\prime }(I_1)}{Q(I_1)}\frac{H^{\prime }(t_1)}{H(t_1)}=0, \end{aligned}$$

16 $$\begin{aligned}&-\frac{F^{\prime \prime }(t_1)}{F(t_1)}+ \frac{K^{\prime \prime }(t_1)}{K(t_1)}+2aI_1\frac{Q^{\prime }(I_1)}{Q(I_1)}\frac{H^{\prime }(t_1)}{H(t_1)}\frac{K^{\prime }(t_1)}{K(_1)} +a^2\left( I_1 \frac{H^{\prime }(t_1)}{H(t_1)} \right) ^2\frac{Q^{\prime \prime }(I_1)}{Q(I_1)} \nonumber \\&\quad + aI_1\frac{Q^{\prime }(I_1)}{Q(I_1)}\frac{H^{\prime \prime }(t_1)}{H(t_1)}>0, \end{aligned}$$

17 $$\begin{aligned}&\frac{D^{\prime }(r_1)}{D(r_1)}+aI_1\frac{Q^{\prime }(I_1)}{Q(I_1)}\frac{D^{\prime }(r_1)}{D(r_1)}-(1-a)I_1\frac{Q^{\prime }(I_1)}{Q(I_1)}S^{\prime }(0)<0, \quad \text {and} \end{aligned}$$

18 $$\begin{aligned}&\frac{D^{\prime }(r_1)}{D(r_1)}+aI_1\frac{Q^{\prime }(I_1)}{Q(I_1)}\frac{D^{\prime }(r_1)}{D(r_1)}+(1-a)I_1\frac{Q^{\prime }(I_1)}{Q(I_1)}S^{\prime }(0)>0. \end{aligned}$$

Cryptic ESSs are a possibility and may constitute a local ESS alongside conspicuous solutions and their stability conditions are generally simpler than those of (15) through to (18). Cryptic solutions with $t_1>0$ are local ESSs if and only if in addition to (18), (15) and (16) hold. The cryptic solution (0, 0) is a (local) ESS if in addition to (18) inequality

19 $$\begin{aligned} \frac{F^{\prime }(0)}{F(0)} - \frac{K^{\prime }(0)}{K(0)}-aI_1\frac{Q^{\prime }(I_1)}{Q(I_1)}\frac{H^{\prime }(0)}{H(0)}>0 \end{aligned}$$

holds.

## An illustrative example

In this section we demonstrate that the model of Broom et al. (2006) can be extricated from two restrictive conjectures that were made upon its publication. In particular, we will demonstrate that both positive and negative combinations of aposematic traits can achieve locally evolutionarily stable outcomes and further that for given level of conspicuousness there can be more than one optimal levels of toxicity. The majority of empirical (and model-based studies) suggest that aposematic traits co-evolve so that increased conspicuousness is coupled with increased unpalatability. Although this is a sensible assumption to make (more conspicuous prey are expected to suffer higher predator attack rates hence necessitating increased levels of defence), we will show that this is not entirely accurate. To showcase our findings we will apply the conditions for evolutionary stability to functional forms that are biologically plausible and mostly similar to those used in Broom et al. (2008) but for which predator attack probability is more sensitive to aversive information. For such a choice of functions the conception that increased conspicuousness necessarily leads to increased rate of attack is less relevant, since conspicuous and aversive prey have small chance of being attacked. The section is made up of two parts: in the first we demonstrate the general process for solving the conditions for (local) evolutionary stability and propose a partitioning of the parameter space from which emerge three distinct types of solution. Choices of parameters are made from within each partition and explicit continua of evolutionarily stable solutions are depicted (second subsection) in the strategy space .

### Procedure for finding local ESSs

We begin by assigning examples of functions to the general forms introduced in Sect. 2 as in Broom et al. (2008), but with *Q* now given as

20 $$\begin{aligned} F(t):= & {} f_0 e^{-ft}; \quad H(t):= t-t_c; \quad K(t):= \frac{k_0}{1+kt}; \quad L(r) = D(r); \nonumber \\ S(x)= & {} max(1-vx,0); \quad \lambda = 0; \quad Q(I):={\left\{ \begin{array}{ll} q_0, \,\, \text {for} \,\, I\le 0 \\ q_0e ^{-qI^2} \,\, \text {for} \,\, I > 0. \end{array}\right. } \end{aligned}$$

As is known from Broom et al. (2006) - see Appendix I also - only cryptic solutions are possible for $I\le 0$. The exact extension of *Q* on this domain is therefore not crucial and we have used the simplest form possible to minimise additional analysis and maintain our focus on conspicuous solutions. The form on $I > 0$ is such that increasingly aversive types are attacked less and less according to a *Gaussian* drop off. This form is to be contrasted with that used in Broom et al. (2008), where the exponent in *Q* involved a linear power in *I*.

Indeed, Darst et al. (2006) studied different genera of the *Dendrobatidae* (poison frog) family and observed that the most conspicuous morphs are the least toxic, while the least conspicuous ones were the most toxic. It was suggested therein that once aposematism has become established in a population of prey that the aposematic traits become decoupled so that arbitrary combinations of these can provide optimal protection against predation and indeed that potentially costly unprofitability may be traded off in favour of bright colouration so that optimal investment in secondary defence will diminish when more cost-effective conspicuousness evolves as a primary defence. Notably, similar observations were made by Wang (2011), who looked at different populations of the *Oophaga granulifera* species within the *Dendrobatidae* family and considered the mechanisms proposed by Darst et al. (2006) to justify his findings.

Darst et al. (2006) were the first to uncover negatively correlated aposematic traits in nature and indeed the first to ever provide a sound explanation for this (using a differential costs analysis based around optimising energy expenditure to reduce predator attack rate). We presently recover solutions in which aposematic traits appear decoupled and negatively correlated, with the underlying mechanism detailed in terms of a exact mathematical framework. In particular, we suggest that traits are selected so as to optimise fitness in the sense of the conditions detailed in the previous section, which we presently solve for the functional forms provided here. Substitution of (20) into (15) provides an explicit expression for the *t*-equilibrium curve in terms of general rate of detection

21 $$\begin{aligned} -f +\frac{k}{1+kt_1}+ 2\alpha D^2(r_1)(t_1-t_c)=0, \end{aligned}$$

where the quantity $\alpha = aqN^2/n^2$ has been introduced and it is assumed here that $I_1 > 0$. The case $I_1\le 0$ is discussed in Appendix I and primarily in the context of crypsis. For the remainder of the discussion, changes in $\alpha$ are attributed to changes in the fraction *N*/*n* representing the relative proportion of prey to predators, with *a* and *q* held fixed (the latter being an inherent property of the predator).

For (21) we note two interesting facts: first, for given level of conspicuousness there can be (at most) two associated values of toxicity at which equilibrium in the *t*-direction can be achieved. Second, the *t*-equilibrium curve can be both increasing and decreasing with increasing conspicuousness and changes in its monotonicity occur on vertices at which the tangent to the curve is vertical. We can see the first by noticing that with appropriate scaling (21) amounts to

22 $$\begin{aligned} t_1^2 + A(r_1)t_1 + B(r_1)=0, \end{aligned}$$

with

$$\begin{aligned} A(r_1) = -\frac{f}{2\alpha D^{2}(r_1)}+\frac{1}{k}-t_c \qquad \text {and} \qquad B(r_1) = \frac{1}{2\alpha D^{2}(r_1)}\left[ 1-\frac{f}{k} \right] -\frac{t_c}{k}. \end{aligned}$$

Clearly for given $r_1$ the LHS of (22) is a second-order polynomial in $t_1$, whose roots

23 $$\begin{aligned} t_1(r_1) = \frac{-A(r_1)\pm \sqrt{A^2(r_1)-4B(r_1)}}{2} \end{aligned}$$

correspond to the optimal level(s) of toxicity for given level of conspicuousness. In particular, the *t*-equilibrium curve can be identified with the set $\{(r_1,t_1): r_1 >0, t_1=t_1(r_1) \}$, which is generated by plotting the roots of (22) for all values of $r_1>0$. Expression (23) provides insight into the relationship of evolutionarily stable aposematic traits but is cumbersome to implement in the numerical analysis.

For the second fact, we apply the IFT expression (14) to (21) - it should be evident that this indeed satisfies the necessary conditions for the theorem - and deduce that the slope at any point $(r_1,t_1)$ of the *t*-equilibrium curve is given by

24 $$\begin{aligned} \frac{4\alpha D(r_1)D^{\prime }(r_1)(t_1-t_c)}{\left( \frac{k}{1+kt_1}\right) ^2-2\alpha D^{2}(r_1)}. \end{aligned}$$

Substitution of (21) into the denominator given above yields the equivalent expression

25 $$\begin{aligned} \frac{-4 \alpha }{f}\frac{D(r_1)D^{\prime }(r_1)(t_1+1/k)^2(t_1-t_c)^2}{t^2+2t_1 \left( \frac{1}{k}-\frac{1}{f}\right) +\frac{t_c}{f}+\frac{1}{k^2}-\frac{1}{fk}}. \end{aligned}$$

The numerator of the fraction in (25) is strictly negative (except at $t_1=t_c$), whereas the sign of the denominator is not restricted in this manner. Indeed, the denominator is a concave-up, second-order polynomial which has two real roots (providing $b<1$) that are given by

26 $$\begin{aligned} T_{SIGN}^{\pm } = \frac{1}{f}-\frac{1}{k} \pm \frac{1}{f}\sqrt{1-b}, \quad \text {where} \quad b:=f\left( \frac{1+kt_c}{k} \right) . \end{aligned}$$

Clearly if $b=1$ the polynomial has one root only (see Figs. 3b and 4b), while if $b>1$ the polynomial is strictly positive. This implies that expression (26) provides a natural partitioning of the parameter space with respect to *b* (at $b=1$) so that there are two distinct descriptions: $b<1$ and $b>1$ with $b=1$ admitting a border-line case. In light of (20) it is clear that for given level of toxicity increasing values of *f* are associated with higher reductions in fecundity, while increasing values of *k* are associated with reduced likelihood of an attack resulting in capture. Therefore *b* can be interpreted as an honest measure of prey sensitivity to investment in toxicity such that prey individuals with $b<1$ can be though of as *t*-insensitive, while those with $b>1$ are *t*-sensitive. Parameters *f*, *k* and $t_c$ with $b \ge 1$ correspond to instances when the polynomial on the denominator of (26) is positive and where the *t*-equilibrium curve is decreasing. In the first case, the strategy space is partitioned so that the equilibrium curve is decreasing whenever $0<t_1<T_{SIGN}^{-}$ or $t_1>T_{SIGN}^{+}$ and increasing when $T_{SIGN}^{-}<t_1<T_{SIGN}^{+}$. Intersections of the *t*-equilibrium curve with the horizontal lines $T_{SIGN}^{\pm }$ are realised as vertices at which a line tangent to the curve is vertical. This is where the branches of the curve described in (23) meet, which can either be achieved at an intersection with $T_{SIGN}^{-}$ where the curve exhibits a local minimum in the *r*-direction ($r_{min}$-type vertex) or at an intersection with $T_{SIGN}^{+}$, where the curve exhibits a local maximum in the *r*-direction ($r_{max}$-type vertex).

Suitable substitution of *f* in terms of *b*, *k* and $t_c$ allows us to re-write (26) as

27 $$\begin{aligned} T_{SIGN}^{\pm } = \left( t_c+\frac{1}{k}\right) \left( \frac{1\pm \sqrt{1-b}}{b} \right) -\frac{1}{k}. \end{aligned}$$

This reformulation indicates that $T_{SIGN}^{-}$ assumes ever-increasing values over the interval $\big (0.5t_c-0.5/k,t_c \big ]$ as *b* increases, while $T_{SIGN}^{+}$ assumes ever-decreasing values over the interval $[t_c,+\infty )$ as *b* increases. In particular, this shows that the width of the region of increasing solutions $(T_{SIGN}^{-},T_{SIGN}^{+})$ is greatest for $b \approx 0$ and least for $b \approx 1$ and shrinks monotonically as *b* increases in between these end values. Furthermore, since $T_{SIGN}^{-}<t_c$ for all choices of *b*, the equilibrium curve cannot exhibit $r_{min}$-type vertices in the aversive region, so that only $r_{max}$-type vertices can be expected for $b<1$.

Thus far, we have established an effective partitioning of the parameter space and have indicated how solutions may vary within these. However, in order to effectively solve (21) we proceed with the following re-arrangement

28 $$\begin{aligned} \frac{1}{D^{2}(r_1)} = \frac{2\alpha }{f}\frac{(t_1+1/k)(t_1-t_c)}{t_1-\frac{1}{f}+\frac{1}{k}}, \end{aligned}$$

which is convenient because solutions can be understood as intersections of the $r_1$-dependent LHS and the $t_1$-dependent RHS. Using $f=bk/(1+kt_c)$ the latter reads

29 $$\begin{aligned} \frac{1}{D^{2}(r_1)} = \frac{2\alpha (1+kt_c)}{bk}\frac{(t_1+1/k)(t_1-t_c)}{t_1-t_{*}}, \quad \text {with} \,\,t_{*} := \frac{1}{k}\left( \frac{1}{b}-1 \right) +\frac{t_c}{b}. \end{aligned}$$

It is evident from the definition of $t_{*}$ that we have recovered the same partitioning of the parameter space at $b=1$. Indeed, if $b<1$ then $t_{*}>t_c$ and the denominator is zeroed at a value greater than $t_c$, while the opposite is true for $b>1$. The critical case $b=1$ is simpler, since $t_{*}=t_c$ and the expression on the RHS of (29) is linear in $t_1$.

Strategies on the *t*-equilibrium curve (21) are *t*-stable if they satisfy (16), which in conjunction with (21) read

30 $$\begin{aligned} \left( \frac{k}{1+kt_1} \right) ^2 - \frac{a}{t_1-t_c} \left( f-\frac{k}{1+kt_1} \right) >0. \end{aligned}$$

Under the assumption that $t_1>t_c$ this is analogous to

31 $$\begin{aligned} t_1^2 + \left( -\frac{1}{af}-\frac{1}{f}+\frac{2}{k} \right) t_1 - \frac{1}{fk}+\frac{1}{k^2} + \frac{t_c}{af}<0. \end{aligned}$$

Since the LHS is concave-up, the inequality can only be satisfied if the parabola has two distinct real roots and $t_1$ is chosen to lie between these. Indeed, we require that the discriminant of the parabola in (31) is strictly positive, which amounts to

32 $$\begin{aligned} a^2 +2a (1-2b) +1>0, \end{aligned}$$

Once again, the parabola in *a* on the LHS of (32) is concave-up and there are three cases to consider depending on whether its discriminant is negative, zero or positive. The discriminant is given by $16b^2(1-1/b)$ and so if $b<1$, all choices of $a \in [0,1]$ give rise to a stable region through (34). If $b=1$ all values of $a \in [0,1)$ will yield a stable region, while for $b>1$ values of $a\in [0,a^-(b))$ work. Notice that we have labelled

33 $$\begin{aligned} a^{\pm }(b):=2b-1 \pm 2b\sqrt{1-1/b} \quad \text {for} \quad b>1, \end{aligned}$$

as the roots of the polynomial in (32). Values of *a* that yield a stable region should in principle also be drawn form the interval $(a^{+}(b),1]$, but it is clear that $a^{+}(b)>1$ for $b>1$. Furthermore, the smaller root $a^{-}(b)$ is decreasing over $b>1$ with $a^{-}(1^+) \approx 1$ and $a^{-}(b)\approx 0$ for $b \gg 1$. It is also self-evident that no *t*-equilibrium strategy can be stable in the *t*-direction whenever $b>1$ and $a\in [a^{-}(b),1]$. In conclusion, given an appropriate choice of $f,k,t_c$ and $\alpha$ the value of $b=f(1+kt_c)/k$ is such that when it is below unity *t*-stable strategies can resist invasion against mutant groups of any size, whereas if it is above unity *t*-stable strategies can withstand invasion against mutant groups of maximum size $a^{-}(b)$. Arguably, choices of *f*, *k* and $t_c$ corresponding to $b=1$ are non-generic.

**Fig. 1:**
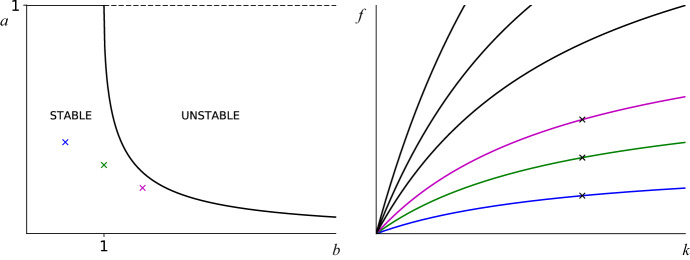
On the left (figure **a**) are shown the sizes *a* of mutant groups, against which the residents can withstand invasion (along the *t*-direction) for given choice of *b*. A *t*-stable strategy with *f*, *k* and $$t_c$$ giving $$b\ge 1$$ have a maximum associated mutant group size of $$a^{-}(b)$$ as described in (33) - which decreases down to zero as *b* increases - whereas *t*-stable strategies with $$b<1$$ can withstand invasion against mutant groups of all sizes. The three x marks blue, green and magenta have coordinates (*b*, *a*) given by (0.5, 0.4), (1, 0.3) and (1.5, 0.2) respectively. Figure **b** on the right shows the *b*-level curves of the map $$(f,k) \mapsto f\big (\frac{1+kt_c}{k} \big )$$, with $$t_c=0.5$$ (this value is used throughout to generate Figs. 3, 4, 5 and 6). *x* marks correspond to $$ b = 0.5, b = 1$$ and $$b =1.5 $$ with $$(f, k) = (0.5, 2), (f, k) = (1, 2)$$ and $$(f, k) = (1.5, 2)$$

In particular, we have established that for the appropriate choices of *a* and *b* described above (see Figure (1) also) a given *t*-equilibrium strategy $(r_1,t_1)$ is stable in the *t*-direction providing that $T_{STAB}^{-}<t_1<T_{STAB}^{+}$, where

34 $$\begin{aligned} T_{STAB}^{\pm } = \frac{1}{2f}\left( \frac{1}{a}+1\right) -\frac{1}{k}\pm \frac{1}{2af}\sqrt{a^2 + 2a(1-2b)+1} \end{aligned}$$

are the roots of the polynomial on the LHS of (31). These roots provide upper and lower bounds to the *t*-stable region and are realised as horizontal lines in the strategy space (as they do not depend on $r_1$). In particular, points $(r_1,t_1)$ on the *t*-equilibrium curve with $T_{STAB}^{-}<t_1<T_{STAB}^{+}$ are stable against invasion from mutants (of maximum group size determined by *b*) that are more or less toxic, while those outside of this region are unstable.

It can be shown that the upper bound $T_{STAB}^+$ shrinks as the size of the invading mutant group grows. Indeed

$$\begin{aligned} \partial _{a}T_{STAB}^+ = -\frac{1}{2a^2f}\big (1+\sqrt{p(a)}\big )+\frac{1}{2af\sqrt{p(a)}}(1+a-2b), \end{aligned}$$

where we have used the shorthand $p(a):=a^2+2a(1-2b)+1$. Clearly, the expression on the RHS is negative for choices $b>1$ and $a \in [0,a^{-}(b))$, while also for the non-generic case $b=1$ and $a\in [0,1)$. For choices $b<1$ and $a\in [0,1]$ we consider the re-scaled inequality

35 $$\begin{aligned} 2a^2f\sqrt{p(a)}\partial _{a}T_{STAB}^{+}<0 \quad \Leftrightarrow \quad 4a^2b^2(1-1/b)<0, \end{aligned}$$

which holds true. Using similar reasoning for the lower bound $T_{STAB}^{-}$

36 $$\begin{aligned} 2a^2f\sqrt{p(a)}\partial _{a}T_{STAB}^{-}<0 \,\, \Leftrightarrow \,\, 4a^2b^2\big (1-1/b \big )<0, \end{aligned}$$

we conclude that for $b<1$ and $a\in [0,1]$ the term $T_{STAB}^{-}$ is shrinking with growing mutant group size *a*, while it increases for growing *a*, whenever $b>1$ and $a\in [0,a^{-}(b))$. Interestingly, for non-generic choices $b=1$ and $a\in [0,1)$ the term $T_{STAB}^{-}$ is constant over all mutant group sizes.

**Fig. 2:**
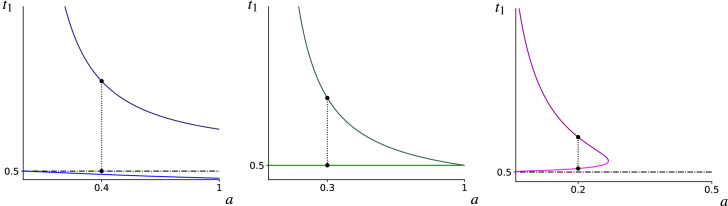
Plots containing $T_{STAB}^{+}$ (top curves) and $T_{STAB}^{-}$ bottom curves versus *a* are shown (see (34)), where $t_c=0.5$ (dashed horizontal lines) has been used. The specific values of *b* used in Figs. 2a, b and c have been generated using the values of *f* and *k* that are indicated by the x marks in Fig. 1b. The x marks in Fig. 1a indicate the values of *a* at which the dotted vertical line segments are drawn (their lengths indicating the width of the stable bands in Fig. 3). Notably the monotonicities of the curves are in agreement with conditions (35) and (36). In particular, Fig. 2a contains curves $T_{STAB}^{\pm }(a) = \frac{1}{a} +0.5 \pm \frac{1}{a} \sqrt{a^2+1}$, line drawn at $a=0.4$. Figure 2b shows $T_{STAB}^{\pm }(a) = \frac{1}{2a} \pm \sqrt{a^2-2a+1} 2a$, with $T_{STAB}^{-}=t_c$, dotted line drawn at $a=0.3$. Figure 2c shows $\frac{1}{3a}-\frac{1}{6}\pm \frac{1}{3a}\sqrt{a^2-4a+1}$ with the dotted line drawn at $a=0.2$

Stability in the *r*-direction is guaranteed by condition (17). With the current functional forms in place, stability against less conspicuous mutants is guaranteed by

37 $$\begin{aligned} -\frac{D^{\prime }(r_1)}{D(r_1)} + 2\alpha D(r_1)(t_1-t_c)^2 \left[ D^{\prime }(r_1)+v\left( \frac{1}{a}-1 \right) D(r_1) \right] >0 \end{aligned}$$

while stability against more conspicuous mutants holds when

38 $$\begin{aligned} -\frac{D^{\prime }(r_1)}{D(r_1)} + 2\alpha D(r_1)(t_1-t_c)^2 \left[ D^{\prime }(r_1)-v\left( \frac{1}{a}-1 \right) D(r_1) \right] <0. \end{aligned}$$

### Explicit examples of evolutionarily stable outcomes

**Fig. 3:**
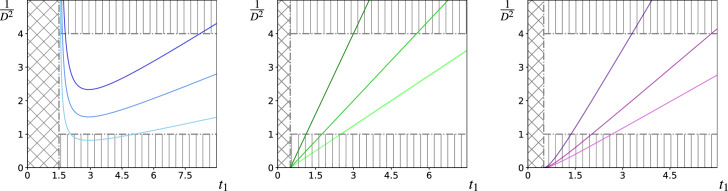
Plots show variation in ${\varvec{\alpha }}$ of the RHS of (28) for the regimes $b<1$ (left), $b=1$ (centre) and $b>1$ (right), such that curves with values of $1/D^2$ for given level of $t_1$ correspond to a higher value of $\alpha$. Only sections of curves contained inside the unshaded region correspond to real ESSs and there are two grey-shaded regions that are empty of solutions: (i) the diagonally-checkered vertical strips, on which the RHS of (29) is negative; (ii) the vertically-striped horizontal sections, on which the LHS of (29), i.e. $1/D^{2}(r_1)$ assumes values outside of the admissible range (1, 4]. **a** Values $f=0.5$, $k=2$, $t_c = 0.5$ and $\alpha = 0.035, 0.065, 0.1$ have been used; curves with $\alpha < 0.042$ have minima inside the bottom grey region, curves with $0.042\le \alpha \le 0.171$ have minima inside the unshaded region and curves with $\alpha >0.171$ are entirely contained in the top grey-shaded region (see Appendix II also). **b** Values $b=1$, $f=1$, $k=2$, $t_c = 0.5$ and $\alpha = 0.25, 0.4, 0.8$ are used and curves with $\alpha >4$ (not shown) would be entirely outside the unshaded region. **c** Values $b=1.5$, $f=1.5$, $k=2$, $t_c = 0.5$ and $\alpha = 0.4, 0.6, 1.2$ have been used and all values of $\alpha$ yield solutions

**Fig. 4:**
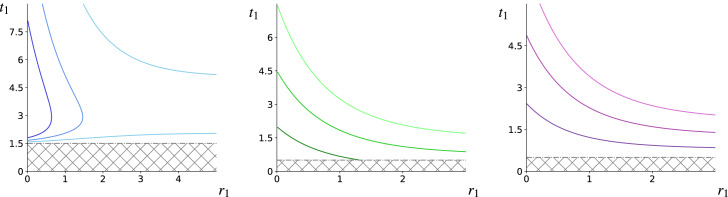
Plots show curves of *t*-equilibrium that result from the root-finding process illustrated in Fig. 3. The diagonally-checkered vertical strips of Fig. 3 are seen here as horizontal grey-shaded strips. **a** The equilibria are disjointed for small values of $\alpha$ and join together at a local $r_{max}$, which shifts toward lower conspicuousness until undefined at $r_1<0$. (b) & (c) Equilibrium toxicity shrinks with increasing levels of $r_1$ and $\alpha$

In this subsection we make use of all results established thus far by assigning specific functional form to the detection rate *D*(*r*) and choosing parameters within the partitions introduced in the previous subsection. In particular, we consider

39 $$\begin{aligned} D(r) = \frac{d_0}{d_0+(1-d_0)e^{-r}} \end{aligned}$$

and without much loss in generality take $$d_0=1/2$$ and $$ t_c=1/2$$. It remains for us to constrain the remaining parameters $$\alpha $$, *a* and *v* so as to explore a suitable range of admissible solutions. Henceforth, specific plots are produced by making the appropriate choice of parameters for a limited set of equalities and inequalities. Indeed, in Sect. 2 it was made clear that local ESSs are those subsections of the *t*-equilibrium curve (defined through equality (15)) that are stable in the in the *t*-direction and in the *r*-direction (inequalities (16), (17) and (18)) and thus considerable emphasis is placed here in exploring the various forms the *t*-equilibrium curve can assume (with $$b<1$$, $$b=1$$ and $$b>1$$ - see Fig. 4) prior to identifying explicit examples of local ESSs. In Sect. 3.1 it was proposed that the *t*-equilibrium curve can be generated by finding intersections of the $$r_1$$-dependent LHS and the $$t_1$$-dependent RHS of equalities (28) or (29), a process which is illustrated in Fig. 3.

We proceed by direct substitution of *D* into the *t*-equilibrium condition, which reads

40 $$\begin{aligned} -f +\frac{k}{1+kt_1}+2\alpha \frac{t_1-0.5}{(1+e^{-r_{1}})^2}=0 \end{aligned}$$

and is generated by interpreting it as an intersection of the LHS with the RHS in the equality below

41 $$\begin{aligned} (1+e^{-r_1})^2 = \frac{2\alpha }{f} \frac{(t_1+1/k)(t_1-0.5)}{t_1-t_{*}}. \end{aligned}$$

Figure 3 shows plots of the RHS of (41) as a function of the resident toxicity for three different regimes of prey sensitivity $b=0.5$ (blue), $b=1$ (green) and $b=1.5$ (magenta) and for different levels of predation pressure $\alpha$. The values for *f*, *k* and $t_c$ used in the plots of Figs. 3a, b and c correspond to the x marks in Fig. 1b. The same parameter values are also used in Fig. 4, which should be viewed in tandem with Figs. 3 since their colour-coding is common (i.e. a curve in Fig. 3 and a curve in Fig. 4 with the same colour represent outcomes that are identical in terms of the parameters used to generate them). Solutions to (41) are realised in Fig. 3 as intersections (not shown) of the coloured curves and the horizontal lines $LHS=c$ in the unshaded region $c \in (1,4]$. Intersections with $c=4$ correspond to cryptic *t*-equilibrium points with $r_1=0$ while those with $c=1^{-}$ correspond to very bright equilibrium points with $r_1 \gg 1$. Intersections with ever-decreasing values of *c* in this interval generate a smooth curves of *t*-equilibrium parametrized with increasing $r_1$ shown as plots over the strategy space in Fig. 4. Generally, Figs. 3 and 4 are indicative of the partition-sensitive behaviour introduced in Fig. 1 and discussed thus far.

As explained earlier, the *b*-partitioning of the parameter space reflects prey sensitivity to investment in toxicity, such that two distinct regimes are understood: prey with $b<1$ are *t*-insensitive, while prey with $b>1$ are *t*-sensitive ($b=1$ is a non-generic description that can be explained using the remaining cases). This means that for given level of investment in toxicity the latter benefit less on account of a comparatively lower rate of reproduction (lower *f*) and/or a lower level of protection against potentially-lethal attacks (higher *k*). The differences between the two regimes are manifest in the curves of *t*-equilibrium, which we explore by varying the level of predation pressure (see Fig. 4) in these. Comparing Figs. 4a and 4c one observes that while *t*-insensitive prey exhibit both increasing and decreasing correlation among aposematic traits (providing predation pressure is moderate-high), *t*-sensitive prey with $b>1$ allow for negatively correlated traits only.

Having explored the nature of the *t*-equilibrium curves, we proceed to giving examples of when sections of these can also constitute local ESSs. That is, we are interested in sections of the *t*-equilibrium curve that are stable in the *t*-direction, namely that are contained within $T_{STAB}^{-}<t_1<T_{STAB}^{+}$ (with $T_{STAB}^{\pm }$ given as in (34)). Furthermore, we require that the *t*-equilibrium strategies are stable against invasion from equally toxic but less conspicuous mutant groups, or rather, that the *t*-equilibrium curve is contained within the region defined by the inequality

42 $$\begin{aligned} -\frac{1}{1+e^{r_1}} + 2\alpha \frac{(t_1-0.5)^2}{(1+e^{-r_1})^2}\left[ \frac{e^{-r_1}}{(1+e^{-r_1})} + v\left( \frac{1}{a}-1\right) \right] >0. \end{aligned}$$

Similarly, stability against invasion from equally toxic but more conspicuous mutant groups is guaranteed by keeping the *t*-equilibrium curve within the region defined by

43 $$\begin{aligned} -\frac{1}{1+e^{r_1}} + 2\alpha \frac{(t_1-0.5)^2}{(1+e^{-r_1})^2}\left[ \frac{e^{-r_1}}{(1+e^{-r_1})} - v\left( \frac{1}{a}-1\right) \right] <0. \end{aligned}$$

**Fig. 5:**
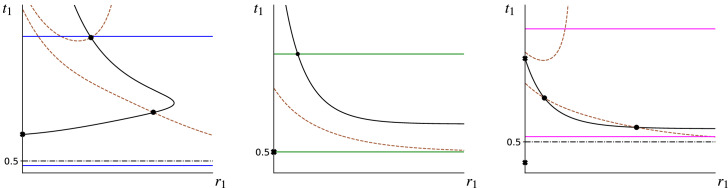
All three figures contain plots of local ESSs in strategy space as subsections of the *t*-equilibrium curve (40) shown as black solid lines and partitioned by black markers (filled X marks indicate cryptic solutions). The dashed-dotted lines mark the natural cut-off at $t_c=0.5$, while the dashed lines represent the zero level sets of the LHS of (42) and (43). In **a** we have set $b=0.5, a=0.4, f=0.5, k=2, \alpha =0.0445$ and $v=0.04$. The solid curve has an $r_{max}$-type vertex at $t_1=T_{SIGN}^{+} = 1.5+\sqrt{2}$ and unique cryptic ESS at the aversive level $t_1 \approx 1.601$. In **b** we have set $b=1,a=0.3, f=1, k=2, \alpha =0.3$ and $v=0.4$. The section of the *t*-equilibrium curve that lies under the upper horizontal solid line and above the brown dashed line consists of a decreasing continuum of local ESSs; $t_1 = 1/2$ is the unique cryptic ESS, although this may be non-generic. In **c** we have set $b=1.5, a=0.2, f=1.5, k=2, \alpha = 1.6$ and $v=0.05$. The section of the solid black curve in between the lower and upper dotted lines consists of a decreasing continuum of local ESSs, the section under the lower curve does not contain ESSs, while the last section that is once more in between the middle and top lines consists of a second decreasing continuum of ESSs. The intersection of the equilibrium curve with $r_1=0$ is an aversive cryptic ESS, while $t_1=1/6$ admits a non-aversive cryptic solution

Important examples of ESSs are shown in Fig. 5, which constitute distinct realisations emerging from the choices for parameters *a*, *b*, *f*, *k* and $t_c$ that are indicated by the x marks in Fig. 1; additional choices for *a* and *v* are specified in the caption. In Fig. 5a, the region below the bottom brown curve is *r*-unstable from the left and the region above the top brown curve is *r*-unstable against mutations from the right, implying that the section of the *t*-equilibrium inbetween these two curves is *r*-stable. The *r*-stable subsection of this that is contained within the blue solid lines (as indicated by the solid markers) is also *t*-stable and therefore contains local ESSs, whereby traits can either be positively or negatively correlated. There are two intersections of the *t*-equilibrium curve with the vertical axis $r_1=0$. The first is not shown and is not a cryptic ESS as it is *t*-unstable, while the other intersection (shown) is an aversive cryptic solution as it satisfies *r*-stability from the right (below the top brown curve) and is *t*-stable (within the blue solid lines). Since $f=0.5<2=k$ it follows that the origin is not an ESS; there are no further cryptic solutions since the LHS of (I.2) gives $t_1 = 1.5>t_c$.

Figure 5b is more straightforward to analyse because *r*-stability from the right is everywhere satisfied. The pair of brown dash-dotted lines are the zero level sets of the LHS of (43), so that only the region inbetween these is potentially *r*-unstable. Since the *t*-equilibrium curve is entirely above the top brown curve, the section of this that is below the top solid green line (as indicated by the marker) consists of local ESSs, all of which indicate negative correlation between conspicuousness and defence. The intersection of the *t*-equilibrium curve with $r_1=0$ is not a cryptic solution as it is *t*-unstable. The origin is once more unstable because $f=1<2=k$. The equilibrium provided by (I.2) is equal to 1/2 and is a cryptic solution. This is likely a non-generic outcome, which we omit from Fig. 5b.

In Fig. 5c the top brown dash-dotted curve is the zero level set of the LHS of (43), so that the region above it fails *r*-stability from the right, while the pair of brown curves below this constitute the zero level set of (42) so that the region inbetween them fails the *r*-stability condition from the left. This means that the section of the *t*-equilibrium curve in between the top and middle brown curves is *r*-stable (and contains local ESSs), while the next section (as indicated by the solid markers) below the middle brown curve is *r*-unstable and the last section of the curve above the brown curve is *r*-stable (and contains local ESSs). The intersection of the *t*-equilibrium curve with $r_1=0$ is a cryptic ESS as *r*-stability from the right is satisfied. The origin is not a cryptic solution. The origin is once more not an ESS since $f=1.5<k=2$. However, the equilibrium given by (I.2) with $t_1=1/6$ is a non-aversive cryptic solution that exists alongside the aversive one mentioned above.

## Discussion

This paper builds on the game-theoretical model of Broom et al. (2006) and constitutes an important exploration into its far-reaching implications. We have established that it can be released from some restrictive conjectures that were made upon its publication and hence argue that it is the only model-based study to provide sound justification for some more curious instances of aposematic behaviour observed among species of the *Dendrobatidae* family. In particular, we have shown that evolutionarily stable combinations of aposematic traits can be either positively or negatively correlated (on an across-populations basis) and that for given level of conspicuousness there can exist more than one optimal levels of defence. Our results were clearly demonstrated in Sect. 3, wherein biologically plausible functional forms, similar to, but more elaborate than those considered previously in Broom et al., (2008) were selected. The conditions for local evolutionary stability (presented in generality in Sect. 2) were solved for values of parameters ranging from within three distinct partitions so that cryptic solutions were discussed alongside conspicuous ones.

In addition to the game-theoretical treatments of Leimar et al. (1986) and Broom et al. (2006) that are discussed in the introduction, there exists a range of publications that examine the co-evolution of aposematic traits from the mathematical modelling perspective and within these a variety of driving mechanisms and notions for optimality are considered (see Summers et al. (2015) for a systematic review). Among these, the majority (see for example Speed and Franks (2014), Holen and Svennungsen (2012), Lee et al. (2011), Blount et al. (2009), and Franks et al. (2009)) alludes to the idea that conspicuousness and defence should be positively correlated, with Speed and Ruxton (2007) admitting the only exception to this. The latter suggests that this correlation can be either positive or negative depending on the variation in marginal costs of aposematic display across populations. Although sound and intuitive, the mechanism presented therein is not expounded in the level of detail that is used here, nor are strategies ’assessed’ in terms of their evolutionary stability. Another contribution is that of Svennungsen and Holen (2007), who investigate the possibility of a stable dimorphism using a game-theoretical description that is similar to our own. However, their focus is automimicry and focus on defences as opposed to the joint co-evolution of a defensive and signalling trait.

This paper is the only theoretical study suggesting that negatively correlated aposematic traits (across different populations) could be evolutionarily stable (as mentioned in the introduction claims that Leimar et al. (1986) identified negatively correlated solutions are not correct). It is important to stress that in our study correlations between aposematic traits are made on an across-populations basis. This is neither true among all model-based studies, nor among empirical studies, some of which additionally consider within population variations while others consider variations across species.

An important assumption of our model is that predators learn quickly to avoid prey that are unpalatable and that aversive learning occupies a short investigative period that takes place early on in their life. Insectivorous birds residing in tropical regions may consume hundreds of insects a day and live several years; for the duration of their life they impose mortality based on these early experiences and could closely fit the predator assumptions of Broom et al. (2006) detailed above. There is a considerable volume of evidence in support of this ’fast learner’ assumption, including the field observations of Brower (1969) on blue jay - monarch butterfly systems, the commentary of Mallet (2001) and the observations of Darst et al. (2006) among others, on chickens feeding on poisonous *Dendrobatidae* frogs.

Among empirical studies, the majority observes that aposematic traits are positively correlated. In particular, Arenas et al. (2015), Blount et al. (2012), Maan and Cummings (2012), Vidal-Cordero et al. (2012), Santos and Cannatella (2011), Cortesi and Cheney (2010), Bezzerides et al. (2007) and Summers and Clough (2001) have all observed positive correlations, while Wang (2011) and Darst et al. (2006) observed negative correlations and lastly Daly and Myers (1967) observed no correlation whatsoever. These studies have considered samples of taxa ranging from marine opisthobranchs, wasps, beetles and frogs and while some consider within-population variations, others look at variations across populations and others yet consider different species of a given genus. Prior to the present publication, no model-based approach could provide sound reasoning to support the possibility of negative correlations and indeed we argue that our present contribution is the only study containing causal confirmation that evolutionarily stable combinations of aposematic traits need not be positively correlated (but instead follow a more complex relationship).

The authors of Broom et al. (2006) had originally anticipated that conspicuousness and defence should be positively correlated. Indeed, this result was confirmed by the functional forms considered therein and subsequently in Broom et al. (2008) and is in fact a sensible assertion to make: not only does this appear to be the prevailing pattern in nature but one would expect that more conspicuous prey should be better defended as they are more likely to be attacked. As we have demonstrated, this reasoning is not entirely accurate, especially once aposematism has become established within a population of prey (although it may better apply during the evolutionary transition from crypsis to aposematic signalling) and instances in nature that appear to negate this cannot be ignored. Indeed, using reflectance spectra and toxicity assays on different populations of *Oophaga granulifera* (a species of poison frog) Wang (2011) observed that the less conspicuous morphs were the most toxic, while the most conspicuous ones were the least toxic. Darst et al. (2006) observed a similar effect among different genera of the *Dendrobatidae* family and hence hypothesised that aposematic traits become decoupled so that arbitrary combinations can reduce anti-predator attack rates. This suggestion supports our own conclusion that for given level of conspicuousness the optimal level of toxicity (providing this exists) need not be unique. Furthermore, Darst et al. (2006) propose that potentially costly unprofitability may be traded off in favour of bright colouration so that optimal investment in secondary defence will diminish when more cost-effective conspicuousness evolves as a primary defence.

It should be noted that this differential costs analysis of Darst et al. (2006) is based on the following assumptions: both the production of the signal and production of the defence are physiologically costly, even if no attacks occur on a particular prey individual (by contrast, we assume that only investment in toxicity is physiologically costly); increased conspicuousness is both increasingly physiologically costly and increasingly effective in reducing the likelihood that a predator successfully kills discovered prey; the same assumptions are true of increased investment in the defence. Thus there is a natural trade-off between investment in either signal or defence that leads to the reported negative correlation; although this may provide a sufficient explanation, it may in fact not be necessary, since the same effect can be explained by evolutionary stability considerations alone. A concise explanation of how we retrieved negatively correlated solutions in this paper cannot be given in an equally similar manner; the model at hand is far more elaborate and as has become clear from comparison with Broom et al. (2008) that the particular results retrieved depend strongly on the functional forms chosen. While in Broom et al. (2008) only increasing solutions are possible, the slight modification in *Q* has opened up the possibility for both positively and negatively correlated traits. Indeed, it is notable herein that individuals that are more sensitive to investment in toxicity ($b \ge 1$) exhibit a negative correlation, while less sensitive individuals ($b<1$) can have both positive and negative correlations; such an effect is unlikely to hold generally.

As far as empirical testing of the model goes, this is a difficult process; the model is elaborate and controlling all the parameters involved would admit an unrealistic task for the biologist. Nonetheless, the connection between Broom et al. (2006) and Darst et al. (2006) can be clarified to some extent. In particular, although the latter is most concerned with the effectiveness of choice of strategy on reducing attack by predators, we are more concerned with its contribution to overall prey fitness. Fitness is understood as the number of offspring produced per life cycle and naturally depends on predator-induced mortality, suggesting that the work of Darst et al. (2006) is important, but which should be supported by additional demographic measurements. For example, a population consisting of mainly young prey would indicate high fecundity and predator-induced mortality, while a population consisting mainly of old prey suggests low fecundity and low predation pressure; these data can be cross-examined with strategy considerations by means of toxicity assays and reflectance measurements as in Darst et al. (2006) to establish a connection between strategy and fitness. Such measurements rely on knowledge of average lifespan among camouflaged prey and should be carried out on an across-populations basis; in practice it may be difficult to identify populations of a given species playing a range of different strategies. Finally, it would be interesting to establish whether investment in conspicuousness is physiologically costly; this would require that we find two populations that are equally toxic and differ only in conspicuousness and even so, demographic differences may not be directly related to differences in the fecundity but to the overall fitness instead.

This brief accounting of empirical and model-based studies (and indeed of this present article’s relevance to these) would be incomplete if some inherent difficulties in interpreting empirical findings were not pointed out. For example (i) studies on certain animals may be naturally more/less relevant to a given model and further, (ii) some are carried out on an intrapopulation basis, others on an across-populations and others yet on an across-species basis (Summers et al. 2015). Notably (i) and (ii) limit the number of potentially relevant studies (there are already not that many) to any given model. (iii) It is believed that there are several factors driving aposematism in real systems (such as variation in life history or community structure for example) and the extent of their individual contributions in any given system may not be generally known (see Speed and Ruxton 2007). (iv) Experimentalists use different operational definitions of conspicuousness and toxicity and the specific techniques used for determining these may make it difficult to compare empirical studies to each other, even if the variables in (i) and (ii) are fixed (see chapter 6 of Ruxton et al. 2018).

The paper at hand constitutes a significant advancement to the model of Broom et al. (2006), which to this day is among the leading model-based treatments of aposematism. Nonetheless, there are areas that remain to be explored within this and which we invite the reader to consider. For example, although we provide explicit examples of local ESSs, we do not discuss whether these can be attained through small, selectively advantageous mutations - this property is known as *convergence stability* and local ESSs are by definition resistant to mutations of this type (this omission is also mentioned in Broom et al. 2006). Indeed, it would be of interest to determine whether a population playing an unstable strategy in the locality of the an ESS could eventually converge to the latter. A separate topic which was explored in Broom et al. (2006) but not presently is the extent to which optimal toxicity can be affected by the appearance of an animal and the degree of kin grouping within the population. In fact, the contribution of colouration to prey appearance (and the existence of non-point solutions) has been completely ignored in the present paper so as to avoid unnecessary complexity with regard to studying the co-evolution of conspicuousness and defence.

The model of Broom et al. (2006) can be extended to consider mimicry systems as well as more general co-existence regimes. For example, we may conceive of a scenario wherein the prey population is made up two types (belonging or not to the same species) each playing a different strategy such that the the intention is to determine whether these can co-exist in a certain proportion over the long term. This is a possibility if the prey population is in a state of *stable equilibrium*, whereby the more fast-paced population dynamics are stable and in addition, each type is (locally) evolutionarily stable in the sense of the definition provided in Sect. 2. Stable co-existence is an interesting problem to consider in generality and particularly so because Batesian and automimicry systems are specific examples of this. Indeed, imposing that both types of prey are equally conspicuous and such that one is positively aversive, while the other is attractive describes a mimicry situation. Although the most immediate extension is automimicry, in which both types belong to the same species, our own interest is in Batesian mimicry - this is also more challenging because to each species pertains a different set of functional forms (such as those considered in Sect. 3).The work of Svennungsen and Holen (2007) is particularly relevant to the extension of our own model to automimicry systems; they investigate the possibility of an evolutionarily stable dimorphism within a game-theoretical framework that resembles our own. As mentioned previously, however, our model studies the joint co-evolution of aposematic traits as opposed to aposematic defences in isolation. Finally, whether or not conspicuous signals can be better recollected by predators in not fully understood and so it may be interesting to consider instances in which the rate of recollection is not simply a scalar of the rate of detection, but is instead scaled by a function that increases with prey conspicuousness.

## Non-aversive solutions

The discussion in Sect. 3 concerns aversive strategies with $I_1>0$ and for good reason; the majority of solutions we tend to be interested in are aversive. A non-aversive strategy $(r_1,t_1)$ can either be conspicuous with $r_1>0$ and $0\le t_1\le t_c$ or cryptic with $r_1=0$ and $0\le t_1\le t_c$. It is a general result of Broom et al. (2006) that non-aversive, conspicuous strategies fail (17) and tend to be invaded by the less conspicuous mutant. Therefore, the only possibility for a non-aversive strategy to be evolutionarily stable is for it to be cryptic.

Alongside existing cryptic strategies that are aversive, there are now two additional possibilities for a non-aversive cryptic solution: either $(0,t_1)$ with $0\le t_1\le t_c$ or the origin (0, 0), but not both. In order to determine the conditions that either of these are stable, we should remark that $I_1 \le 0$ and therefore that according to (20) we have $Q^{\prime }/Q = 0$. This assumption suggests that the predator does not distinguish between different levels of toxicity among non-aversive prey and in particular that mutants are attacked at the same rate as the residents. Since $Q^{\prime }/Q$ features strongly in the conditions for stability, these are notably simpler here. Furthermore, we should remark that a cryptic strategy is *r*-stable if it can resist invasion against the more conspicuous mutant. Since this mutant is attacked at the same rate as the associated resident it follows that *r*-stability from the right is trivially true for non-aversive residents since

I.1 $$\begin{aligned} -\frac{D^{\prime }(0)}{D(0)}<0. \end{aligned}$$

Detectability is the only factor influencing marginal differences in fitness so it follows that the more conspicuous mutant admits a clear disadvantage compared with the resident. It also follows that the evolutionary stability of non-aversive strategies is governed by their stability along the *t*-direction.

The origin is a local ESS if the LHS of (21) is greater than zero. This holds true if $f>k$. The strategy $(0,t_1)$ is an ESS if $t_1<t_c$ and it satisfies the equilibrium condition

I.2 $$\begin{aligned} -f+ \frac{k}{1+kt_1} = 0 \,\, \Leftrightarrow \,\, t_1 = \frac{1}{f}-\frac{1}{k}. \end{aligned}$$

As it happens any such equilibrium is also *t*-stable, since (16) amounts to

I.3 $$\begin{aligned} -f^2 + \frac{2k^2}{(1+kt_1)^2}>0 \,\, \Leftrightarrow \,\, t_1 < \frac{\sqrt{2}}{f}-\frac{1}{k}, \end{aligned}$$

which holds true for an equilibrium given as in (I.2). In summary, we deduce that if $f>k$ then the origin is a non-aversive ESS, while if $f<k$ and $1/f-1/k<t_c$ then the LHS of the latter is an ESS.

## More on the root-finding process for $b<1$

The purpose of this supplementary section is to elucidate the root-finding process detailed in Figs. 3 and 4. In particular, we focus on the $b<1$ regime, wherein upon generating the curves of *t*-equilibrium as changes in the parameter $\alpha$, we identified four critical values for this, whose definitions we provide here. The root-finding process is summarised in equalities (28), (29), the RHS of which admit a local minimum. The critical values of $\alpha$ are thus defined with respect to their crossing of the limiting values of the LHS of the same equalities (28) and (29). That is, the local minimum crosses the bottom dash-dotted line in Fig. 3a for a value of $\alpha =\alpha _1$ defined by

II.1 $$\begin{aligned} -f +\frac{k}{1+kT_{SIGN}^{+}}+2\alpha _1D^{2}(\infty )(T_{SIGN}^{+}-t_c)=0. \end{aligned}$$

The same minimum crosses the upper (at value four) dash-dotted line for $\alpha$ given by

II.2 $$\begin{aligned} -f +\frac{k}{1+kT_{SIGN}^{+}}+2\alpha _2D^{2}(0)(T_{SIGN}^{+}-t_c)=0. \end{aligned}$$

From these definitions it is clear that there are three different types of equilibrium depending on which partition $\alpha$ is chosen from $[0,\alpha _1) \cup [\alpha _1,\alpha _2]\cup (\alpha _2,1]$.

In Figs. 3a and 4a the two critical values of $\alpha$ are given by $\alpha _1 = (3-2\sqrt{2})/4$, $\alpha _2 = (3-2\sqrt{2})$. For $\alpha < (3-2\sqrt{2})/4$ the aversive maximum lies below unity (lower dash-dotted line) and the associated *t*-equilibrium curve in 4(a) consists of two disjointed roots in the aversive region that come closer with increasing $r_1$. For $(3-2\sqrt{2})/4<\alpha <3-2\sqrt{2}$ the minimum of 3(a) lies between unity and four and so the equilibrium curve in 4(a) exhibits an $r_{max}$-type vertex in the aversive region. For $3-2\sqrt{2} < \alpha \le 1$ the minimum is above four, which is why in 4(a) the equilibrium curve is not defined.

## Derivatives of the payoff

In this section we provide a number of limit definitions that are supplementary to the discussion of Sect. 2.2. Let *h* be positive and arbitrarily small. By $\partial _{t}P(r,t;r_1,t_1)$ we mean the partial derivative of the mutant payoff with respect to the mutant toxicity *t* for fixed values of the mutant conspicuousness *r* and remaining resident traits ${r_1}$ and ${t_1}$. That is

III.1 $$\begin{aligned} \partial _{t}P(r,t;r_1,t_1) := \underset{|h|\rightarrow 0}{\lim }\frac{P(r,t+h;r_1,t_1)-P(r,t;r_1,t_1)}{|h|}. \end{aligned}$$

Higher order derivatives can be defined in a similar way. Along the *r*-direction the notation and definition are similar, except that precisely at ${r=r_1}$ this quantity is not defined. Instead for ${r=r_1}$ we use the left and right partial derivatives

III.2 $$\begin{aligned} \overset{\leftarrow }{\partial }_{r}P(r,t;r_1,t_1)|_{r=r_1} {:=} \underset{|h|}{\rightarrow }{0}{\lim }\frac{{P(r_1,t;r_1,t_1)}-{P(r_1-|h|,t;r_1,t_1)}}{|h|} \end{aligned}$$

and

III.3 $$\begin{aligned} \overset{\rightarrow }{\partial }_{r} P(r,t;r_1,t_1)|_{r=r_1} {:=} \underset{|h|\rightarrow 0}{\lim }\frac{P(r_1+|h|,t;r_1,t_1)-P(r_1,t;r_1,t_1)}{|h|}. \end{aligned}$$

## Remarks about the implicit function theorem

In this section we wish to make a number of technical clarifications. We begin by stating proving and verbally explaining the following result

IV.1 $$\begin{aligned} \partial _{t_1}\big [\partial _{t}P(r,t;r_1,t_1)|_{r=r_1,t=t_1}\big ]|_{r_1=r_1^*,t_1=t_1^*} = \partial _{t_1t}P(r_1^*,t_1^*) + \partial _{tt}P(r_1^*,t_1^*). \end{aligned}$$

To prove this result we use the limit definition of the derivative provided in Appendix III to show that the LHS = RHS. The LHS involves taking two derivatives. Taking the first amounts to

IV.2 $$\begin{aligned} \partial _{t}P(r,t;r_1,t_1)|_{r=r_1, \, t=t_1} = \underset{|h|\rightarrow 0}{\lim }\frac{P(r_1,t_1+h;r_1,t_1)-P(r_1,t_1;r_1,t_1)}{z}{|h|}. \end{aligned}$$

and taking the second gives the required result

$$\begin{aligned} \begin{aligned}&{\partial _{t_1}\big [\partial _{t}P(r,t;r_1,t_1)|_{r=r_1,t=t_1}\big ]|_{r_1=r_1^*,\,t_1=t_1^*}} \\&\quad =\lim _{h_1 \rightarrow 0}\frac{1}{h_1} \left\{ \lim _{h \rightarrow 0}\frac{P(r_1^*,(t_1^*+h)+h_1;r_1^*,t_1^*)-P(r_1^*,t_1^*+h_1;r_1^*,t_1^*)}{h} \right. \\&\left. \qquad -\lim _{h \rightarrow 0}\frac{P(r_1^*,t_1^*+h;r_1^*,t_1^*)-P(r_1^*,t_1^*;r_1^*,t_1^*)}{h}\right\} \\&\qquad + \lim _{h_1 \rightarrow 0}\frac{1}{h_1} \left\{ \lim _{h \rightarrow 0}\frac{P(r_1^*,t_1^*+h;r_1^*,t_1^*+h_1)-P(r_1^*,t_1^*;r_1^*,t_1^*+h_1)}{h} \right. \\&\left. \qquad -\lim _{h \rightarrow 0}\frac{P(r_1^*,t_1^*+h;r_1^*,t_1^*)-P(r_1^*,t_1^*;r_1^*,t_1^*)}{h}\right\} \\&\quad {= \partial _{t_1t}P(r_1^*,t_1^*) + \partial _{tt}P(r_1^*,t_1^*).} \end{aligned} \end{aligned}$$

In Sects. 2 and 3 we utilised the IFT in two dimensions to argue that the continuum of evolutionary stable traits is such that one can draw a line tangent to this curve (existence). Furthermore the same theorem provides a rule - see (14) - that determines the value of the slope of this tangent. Here we show that the tangent is indeed well-defined in the case of the equilibrium curve in question. We proceed as follows.

From conditions (15) through to (18) it is clear that if evolutionarily stable outcomes exist these are manifest as a subset of the continuum defined by the equilibrium condition ${\partial _{t}P(r,t; r_1,t_1)|_{r=r_1, t=t_1}=0}$, which due to (5) amounts to (15). Note that while the quantity on the RHS of

IV.3 $$\begin{aligned} {\partial _{t}P(r,t;r_1,t_1)}= & {} \frac{F^{\prime }(t)}{D(r)K(t)Q(I)} - \frac{F(t)K^{\prime }(t)}{D(r)K^2(t)Q(I)} \nonumber \\&- aI\frac{H^{\prime }(t)}{H(t)}\frac{F(t)Q^{\prime }(I)}{D(r)K(t)Q^2(I)} \end{aligned}$$

is not differentiable with respect to the mutant conspicuousness trait it is by construction continuous at ${(r,t) = (r_1,t_1)}$ at the resident value ${(r,t)=(r_1,t_1)}$ it is continuous at that value. Evaluating the RHS of the latter at ${(r,t)=(r_1,t_1)}$ gives

IV.4 $$\begin{aligned} {\partial _{t}P(r,t;r_1,t_1)|_{r=r_1,\,t=t_1}}&{=}&{\frac{F^{\prime }(t_1)}{D(r_1)K(t_1)Q(I_1)} - \frac{F(t_1)K^{\prime }(t_1)}{D(r_1)K^2(t_1)Q(I_1)}}\nonumber \\&{- aI_1\frac{H^{\prime }(t_1)}{H(t_1)}\frac{F(t_1)Q^{\prime }(I_1)}{D(r_1)K(t_1)Q^2(I_1)}} \end{aligned}$$

where the RHS of the latter is differentiable with respect to the resident traits ${r_1}$ and ${t_1}$. The set of points satisfying the condition ${\partial _{t}P(r,t;r_1,t_1)|_{r=r_1,\,t=t_1}=0}$ is the same as those satisfying condition

$$\begin{aligned} {\frac{F(t_1)}{D(r_1)K(t_1)Q(I_1)}\partial _{t}P(r,t;r_1,t_1)}|_{r=r_1,\,t=t_1}={0} \end{aligned}$$

and in particular the IFT in two dimensions can be applied directly to

IV.5 $$\begin{aligned} {\frac{F^{\prime }(t_1)}{F(t_1)} - \frac{K^{\prime }(t_1)}{K(t_1)}-aI_1\frac{Q^{\prime }(I_1)}{Q(I_1)}\frac{H^{\prime }(t_1)}{H(t_1)}=0}. \end{aligned}$$
